# Editorial: Extracellular matrix in homeostasis and cancer

**DOI:** 10.3389/fgene.2022.1107969

**Published:** 2022-12-09

**Authors:** Ana Rita Carlos, Valérie Allamand

**Affiliations:** ^1^ cE3c & CHANGE, Faculdade de Ciências, Universidade de Lisboa, Lisboa, Portugal; ^2^ Sorbonne Université-Inserm, Centre de Recherche en Myologie, Institut de Myologie, Paris, France; ^3^ Unit of Muscle Biology, Department of Experimental Medical Science, Lund University, Lund, Sweden

**Keywords:** extracellar matrix, cancer, tissue homeostasis, tissue regeneration, cancer biomarkers, stress and damage

Organs are composed not only of a variety of cells, but also of extracellular matrices (ECMs), which provide a physical scaffold and specialized microenvironment for cells and tissues, and allow biochemical signaling. Particularly since the 1970s, the ECM started to be recognized as a highly dynamic component of tissues and the importance of ECM remodeling was found to play an important role in embryonic development, tissue repair and regeneration. In addition to the key ECM components, namely collagens, laminins, fibronectin, elastin, hyaluronan and proteoglycans, our current understanding of ECM dynamics and its tissue specificity allowed us to expand the list of ECM components, collectively termed the matrisome (Naba et al., 2012). In addition to these “core” components (around 300 in mammals), there is a variety of ECM-related molecules, which include proteins required for ECM remodeling, cytokines, chemokines and growth factors, together revealing the dynamic nature of the ECM.

For this Research Topic, we thought of bringing together contributions on how the ECM plays a critical role in tissue homeostasis and disease, with special emphasis on how ECM remodeling and mutations contribute to cancer.

In recognition of the key importance of ECM in tissue development and homeostasis, there are emerging studies using *in vitro* three-dimensional models, where the ECM composition and structure directly impacts cell proliferation, migration, differentiation or survival, mimicking *in vivo* conditions. In keeping with this notion, Carvalho and others have shown that ECM is able to modulate proliferation of mesenchymal stromal cells (MSC), a multipotent stem/progenitor cell population capable of proliferating and differentiating into a variety of lineages, such as adipogenic, chondrogenic, and osteogenic (Carvalho et al.). Moreover, the authors show that ECM derived from young donors was able to rescue the impaired proliferative and osteogenic potential of aged MSC, therefore highlighting the importance of ECM composition and its potential for therapeutic intervention.

Considering the key role of ECM in maintaining tissue integrity, and promoting tissue repair and regeneration, it is not surprising that when the composition of ECM is altered, for example due to mutations or changes in the levels of ECM components this can also lead to diseases. These changes in the levels of ECM components may be triggered by several endogenous and exogenous insults, that can promote increased oxidative stress and DNA damage, as reviewed by Martins *el al*. In this review, the authors go over how DNA damage and oxidative stress affect the expression and deposition of ECM molecules, and conversely how mutations in the genes that code for ECM components cause an increase in DNA damage and oxidative (Martins et al.). Both circumstances make it challenging for cells and tissues to return to a state of homeostasis, which has an adverse effect on organ and tissue function and may be a trigger for several disorders. An example of these disorders includes cancer, in which cancer cells manipulate a number of elements involved in ECM remodeling to serve their own purposes, promoting cancer cell migration, proliferation, and epithelial-to-mesenchymal transition. In accordance, several studies have revealed the potential of using ECM and associated components as prognostic signatures for different types of cancer. This is the case of *CTHRC1* (collagen triple helix repeat containing 1) and *LRFN4* (leucine rich repeat and fibronectin type III domain containing 4), as prognostic signature in stomach adenocarcinoma (Han et al.), *COL4A6* (collagen IV alpha six chain), *FGA* (fibrinogen alpha chain) and *FSCN1* (fascin actin-bundling protein 1), as potential biomarkers for lung adenocarcinoma (Zeng et al.) or *FN1* (fibronectin), *LAMB4* (laminin beta four chain), *LAMB3* (laminin beta three chain), *DMP1* (dentin matrix acidic phosphoprotein 1), *CHAD* (chondroadherin), and *MMRN1* (multimerin 1, also called elastin microfibril interfacer 4), as a matrisome-related gene signature of head and neck squamous cell carcinoma (Huang et al.).

Altogether these findings stress how crucial ECM is for preserving tissue homeostasis. Improving our understanding regarding the matrisome and other ECM-related molecules will be critical to improve therapeutic strategies in cancer. In addition, and considering that mutations in genes that encode ECM components have been linked to a variety of genetic disorders, such as different neuromuscular diseases, neurodegenerative diseases, Ehlers Danlos Syndromes, and osteogenesis imperfecta, this knowledge will have a wide clinical application.

## References

[B1] Naba A. Clauser K. R. Hoersch S. Liu H. Carr S. A. Hynes R. O. (2012). The matrisome: *In silico* definition and *in vivo* characterization by proteomics of normal and tumor extracellular matrices. Mol. Cell. Proteomics 11, M111.014647. 10.1074/MCP.M111.014647 PMC3322572 22159717

